# Effects of AG490 and S3I-201 on regulation of the JAK/STAT3 signaling pathway in relation to angiogenesis in TRAIL-resistant prostate cancer cells *in vitro*

**DOI:** 10.3892/ol.2014.1795

**Published:** 2014-01-14

**Authors:** VENHAR GURBUZ, ECE KONAC, NURAY VAROL, AKIN YILMAZ, SERHAT GUROCAK, SEVDA MENEVSE, SINAN SOZEN

**Affiliations:** 1 Department of Medical Biology and Genetics, Faculty of Medicine, Gazi University, Ankara 06500, Turkey; 2 Department of Pediatric Infectious Diseases, Faculty of Medicine, Hacettepe University Ankara, Ankara 06100, Turkey; 3 Department of Urology, Faculty of Medicine, Gazi University, Ankara 06500, Turkey

**Keywords:** angiogenesis, apoptosis, JAK and STAT inhibitors, JAK/STAT pathway, prostate cancer

## Abstract

The aim of the present study was to analyze the molecular mechanisms involved in blocking the signaling pathway and the effects of this on the progression of prostate cancer (CaP) cells *in vitro*. LNCaP human CaP cell line was stimulated with interleukin-6 (IL-6) in the presence/absence of Janus kinase (JAK) 2 (AG490), signal transducer and activator of transcription 3 [(STAT3) S3I-201] inhibitors and tumor necrosis factor-related apoptosis-inducing ligand (TRAIL). Cytotoxic activity, the activation of phosphorylated (p)-STAT3 protein, caspase (CASP) 3 activity at protein level, vascular endothelial growth factor (VEGF) A, VEGFC, vascular endothelial growth factor receptor 2, STAT3, matrix metalloproteinase-2, myeloid cell leukemia sequence 1 (MCL-1), CASP8 and CASP9 messenger RNA (mRNA) levels were determined. Morphology and apoptosis were confirmed by DAPI staining and terminal deoxynucleotidyl transferase-mediated dUTP nick-end labeling (TUNEL) assay. IL-6 rapidly induced the phosphorylation of STAT3 in a dose- and time-dependent manner with a peak expression at 3 h at a concentration of 25 ng/ml. In addition, AG490 (50 μM) and S3I-201 (300 μM) inhibited STAT3 activation. Western blotting results revealed that p-STAT3 protein expression decreased significantly with AG490 and S3I-201 treatment in LNCaP cells. AG490 and S3I-201 induced the downregulation of VEGFA, MCL-1 and STAT3 and the upregulation of CASP8 and CASP9 mRNA transcription levels. In addition, the inhibitors increased the level of CASP3 protein. Combinations of AG490- and S3I-201-TRAIL did not result in an increase in this effect. Parallel results were found by DAPI staining and TUNEL assay. To the best of our knowledge, this is the first study to investigate the possible clinical use of AG490 or S3I-201, together with the reduced use of chemotherapeutic agents with high cytotoxicity, for their ability to exert an apoptotic effect, targeting the JAK/STAT3 pathway.

## Introduction

Novel understandings of the early molecular events in prostatic carcinogenesis have emerged that may underlie the genetic and clinical heterogeneity (1). Numerous molecular abnormalities have been previously described in prostate cancer (CaP), including chromosomal loss or gain, gene amplification, mutations leading to the increase or decrease of gene expression levels and changes in the function of proteins. A number of genes associated with inflammation, cell cycle regulation, cell signaling pathways, cell proliferation, steroid hormone metabolism and regulation of gene expression have been implicated (2,3).

The binding of interleukin-6 (IL-6) cytokine family ligands, first to the IL-6 receptor (IL-6R) α and then to the gp130 receptor complex, activates the Janus kinase (JAK)/signal transducer and activator of transcription 3 (STAT3) signal transduction pathway, where STAT3 is important in cell proliferation, differentiation, survival, apoptosis, angiogenesis and tumorigenesis. Circulating serum levels of IL-6 are raised in hormone-refractory CaP patients and evidence from previous cell line studies has suggested that the IL-6R/JAK/STAT3 pathway may be involved in the development of CaP (4,5). Tumorigenesis associated with IL-6 has been attributed to the constitutive or aberrant activation of STAT3 (6). JAK/STAT signaling pathway is well-known to be important in the carcinogenesis of several cell types (7). Previous *in vitro* functional experiments using CaP cell lines have shown that STAT3 is constitutively active in these cell lines and promotes the metastatic progression of CaP (8,9). Furthermore, tyrosine-phosphorylated (p)-STAT3 is observed in 82% of human prostate tumors and expression levels correlate with the Gleason score (8).

Blocking of the gp130 signaling pathway, at the JAK level, may be a useful therapeutic approach against cancer owing to the inhibition of STAT3 activity. JAK2 tyrosine kinase inhibitor tyrphostin AG490 has been widely used as a method of blocking the activation of STAT3 *in vitro* and *in vivo* (10–12). In addition, a number of JAK2 inhibitors have been found to be tolerable with no adverse impact on the quality of life of patients. Therefore, JAK2 inhibitors are crucial in the management of patients with CaP (13).

STAT proteins are cytoplasmic transcription factors that transduce signals from cytokines/growth factors to the nucleus. STAT3, a major member of the STAT family, is involved in various biological cell processes. Therefore, it has become a focus of interest as a new target for cancer therapy similar to that of the JAK proteins (14,15). Previously, activated STAT3 has been shown to promote cell proliferation, metastasis and angiogenesis and to protect tumor cells from apoptosis by regulating associated genes, including Bcl-xL, Bcl-2, Fas, cyclin D1, c-myc, vascular endothelial growth factor (VEGF), matrix metalloproteinase (MMP)-2/-9, myeloid cell leukemia sequence 1 (MCL-1) and survivin (16–19). Abnormalities in the JAK/STAT3 pathway are critical in the oncogenesis of several types of cancer (20) and are involved in the survival, proliferation and metastases of CaP (21–23). Inhibition of STAT3 has been shown to induce apoptosis in CaP cells (13,24). S3I-201 is a chemical probe inhibitor of STAT3 activity and inhibits STAT3:STAT3 protein dimer complex formation and STAT3 DNA binding and transcriptional activities. Furthermore, S3I-201 inhibits growth and induces apoptosis preferentially in tumor cells that contain persistently activated STAT3 (25,26) and in fibrotic kidney disease (27).

VEGF expression has been found to correlate with STAT3 activity in diverse human cancer cell lines. p-STAT3 is a mediator and biomarker of endothelial activation that reports VEGF-vascular endothelial growth factor receptor 2 (VEGFR2) activity (28). Previous studies (28) have provided evidence that the VEGF gene is regulated directly by the STAT3 protein. In addition, STAT3 is a common molecular target for blocking angiogenesis induced by multiple signaling pathways in various types of human cancer. Targeting STAT3 with a small molecule inhibitor blocks hypoxia inducible factor-1 and VEGF expression *in vitro* and inhibits tumor growth and angiogenesis *in vivo* (16,29). Tumor necrosis factor-related apoptosis-inducing ligand (TRAIL) inhibits messenger RNA (mRNA) expression of VEGF, together with matrix metalloproteinase-2 (MMP-2) and tissue inhibitor of matrix metalloproteinases-2 (TIMP-2) in different human glioblastoma cell lines. Thus, the TRAIL system may be regarded as a molecular target to be investigated for innovative therapy of this type of tumor (30). Knockdown of osteopontin, a secreted phosphoglycoprotein, may downregulate MMP-2 and -9 expression, resulting in inhibition of the malignant physiological behaviors of CaP PC-3 cells (31). STAT3 is a mediator of angiogenic as well as antiapoptotic genes. Activation of STAT3 in response to polyamine depletion increases the transcription and subsequent expression of antiapoptotic Bcl-2 and the inhibitors of apoptosis (IAP) family of proteins and thereby, promotes the survival of cells against tumor necrosis factor α-induced apoptosis (32). MCL-1 is a member of the Bcl-2 family, which inhibits cell apoptosis by sequestering proapoptotic proteins, Bim and Bid. MCL-1 overexpression has been associated with the progression of leukemia and numerous solid tumors, including CaP (33). However, the regulatory mechanism for MCL-1 expression in CaP cells remains elusive.

The purpose of the present study was to investigate the inhibitory and apoptotic effects of AG490, S3I-201 and TRAIL combinations on the JAK/STAT3 signaling pathway in a human prostatic carcinoma cell line (LNCaP). Inhibition of the JAK/STAT3 signaling pathway may offer a novel strategy for CaP treatment and as yet, has not been the subject of any study. In addition, the study aimed to assess the biological fate of the LNCaP cells by examining VEGFA, VEGFC, VEGFR2, STAT3, MMP-2, MCL-1 and caspase (CASP) 3, CASP8 and CASP9 gene expression profiles.

## Materials and methods

### Cell line and culture conditions

The LNCaP, IL-6-negative, cell line was obtained from the Marmara University, Faculty of Medicine, Department of Urology (Istanbul, Turkey). The cell line was cultured in Dulbecco’s modified Eagle media (HyClone, Logan, UT, USA) supplemented with 2 mM L-glutamine, 10% heat-inactivated fetal bovine serum, 100 U/ml penicillin G and 100 mg/ml streptomycin (all obtained from HyClone) and kept at 37°C in a humidified incubator with an atmosphere of 5% CO_2_ and 95% air.

### IC_50_ determination and cell proliferation assay

The cells were seeded as 5×10^3^ cells per well in 200 μl complete culture medium containing various concentrations of IL-6 (ranging between 1 and 100 ng/ml; recombinant human IL-6; Raybiotech, Inc., Norcross, GA, USA), AG-490 (ranging between 1 and 100 μM; Bioshop Canada, Inc., Burlington, ON, Canada), S3I-201 (ranging between 1 and 300 μM; Calbiochem, Ukraine) and TRAIL (ranging between 25 and 1,000 ng/ml; ABR; Thermo Fisher Scientific, Inc., Waltham, MA, USA). The cells were incubated for 24, 48 and 72 h, respectively, to determine cytotoxic and apoptotic effects. Cells treated with 0.1% dimethyl sulfoxide served as a solvent control. Each concentration of IL-6, AG490, S3I-201 and TRAIL for each incubation period was repeatedly incubated in four wells to identify the most efficient dose(s) and incubation period(s). Viability IC_50_ values were identified as the concentrations of the chemical agents causing 50% decrease in cell viability. Cytotoxic activity was measured using the water-soluble tetrazolium salt-1 (WST-1) assay (Roche Diagnostics GmbH, Mannheim, Germany), following the manufacturer’s instructions as previously described by Fortmüller *et al* (34). The spectrophotometrical absorbance of the samples was measured at 450 nm in a microplate enzyme-linked immunosorbent assay (ELISA) reader (Spectramax M3; Molecular Devices, LLC., Sunnyvale, CA, USA). Experiments were conducted in triplicate and repeated at least three times. The IC_50_ was set as the values obtained when AG490 and S3I-201 concentrations were decreased to 50% of the control values. The absorbance values were normalized by assigning the value of the parent line in medium without drug to 1.0 and the value of the no-cell control to 0. The most efficient incubation periods and doses of IL-6 and TRAIL were calculated for cell viability. The following IC_50_ values were used for the LNCaP cell line: AG490, 50 μM; and S3I-201, 300 μM.

### Isolated p-STAT3 protein ELISA

LNCaP cells were incubated with 25 ng/ml (optimal effective dose) IL-6 for 3 h, followed by incubation with 300 μM S3I-201 and 50 μM AG490 for various time periods (30 min to 6 h). Cells were washed with ice-cold phosphate-buffered saline and cell pellets were suspended in cell lysis buffer (Cell Signaling Technology, Inc., Danvers, MA, USA) containing 1 mM phenylmethanesulfonyl fluoride and proteinase inhibitor cocktails (Roche Diagnostics GmbH). Cell lysates were incubated on ice and centrifuged for 10 min at 10,000 × g. Protein concentrations were determined in all the cell extracts using the BCA Protein Assay (Thermo Fisher Scientific).

ELISA assay [PathScan^®^ pSTAT Sandwich ELISA (Try 705); Cell Signaling Technology, Inc.] was then performed according to the manufacturer’s instructions. Briefly, standard ELISA procedures were performed using rabbit monoclonal IgG primary antibodies against p-STAT3 with biotin-conjugated anti-mouse IgG as the secondary antibody and horseradish peroxidase-conjugated streptavidin. Quadruplicate assays were performed on each sample and the absorbance at 450 nm was recorded by ELISA reader (Spectramax M3; Molecular Devices, LLC.).

### p-STAT3 protein western blotting

Lysates containing 100 μg protein were mixed with loading buffer with 5% β-mercaptoethanol and heated for 5 min at 100°C. The protein samples were separated using sodium dodecyl sulfate-polyacrylamide gel electrophoresis (SDS-PAGE) and transferred onto nitrocellulose membranes by semi-dry blotting. SDS-PAGE was performed via standard procedures according to the manufacturer’s instructions (Invitrogen Life Technologies, Carlsbad, CA, USA). Membranes were incubated in blocking buffer [Tris-buffered saline (TBS), 0.1% Tween 20 and 5% low-fat dry milk] for 1 h at room temperature, followed by hybridization with anti-p-STAT3 (tyr-705) antibody (1:1,000 dilution; Cell Signaling Technology, Inc.), anti-STAT3 antibody (1:1,000 dilution; Cell Signaling Technology, Inc.) and anti-β-actin antibody (internal control; Santa Cruz Biotechnology, Inc., Santa Cruz, CA, USA) at 4°C overnight. Following three washes in TBS/0.1% Tween 20, the membranes underwent hybridization with a horseradish peroxidase-conjugated secondary antibody rabbit IgG (1:5,000 dilution; Santa Cruz Biotechnology, Inc.) for 1 h at room temperature. Following extensive washing in TBS/0.1% Tween 20, signals were detected by electrogenerated chemiluminescence techniques using SuperSignal West Pico Chemilumiscent Substrate (Thermo Fisher Scientific).

### Quantitative polymerase chain reaction (qPCR) analysis of VEGFA, VEGFC, VEGFR2, STAT3, MMP-2, MCL-1, CASP8 and CASP9 mRNA expression

Total RNA was isolated from each 96-well plate using the High Pure RNA isolation kit (Roche Diagnostics GmbH) according to the manufacturer’s instructions. The yield and quality of the RNA of each sample was determined by measuring the absorbance at 260 and 280 nm using the Nanodrop spectrophotometer (NanoDrop ND-1000; NanoDrop Technologies, Inc., Montchanin, DE, USA). Total RNA (1 μg) was reverse-transcribed in a 20 μl reaction mixture using random hexamers and Transcriptor First Strand cDNA synthesis kit (Roche Diagnostics GmbH) according to the manufacturer’s instructions.

VEGFA, VEGFC, VEGFR2, STAT3, MMP-2, MCL-1, CASP8 and CASP9 mRNA expression levels were measured using qPCR method. Probes and primer sets for each gene were designed at the ProbeFinder Design Assay Center website (https://www.roche-applied-science.com/sis/rtpcr/upl/adc.jsp). PCR primers (exon-exon junction to allow discrimination between cDNA and gDNA) and Universal Probe Library (UPL) numbers are provided in Table I. The 10 ml reaction mixture, prepared in borosilicate glass capillaries, contained 1X LightCycler TaqMan Master reaction mixture (Roche Diagnostics GmbH), 2.5 pmol of each primer, 1 pmol UPL probe, 4 mM MgCl_2_ and 1 μl cDNA. The qPCR assay included a no-template control. PCR reactions were performed in the LightCycler 1.5 instrument (Roche Diagnostics GmbH) using the following denaturing conditions: 95°C for 10 min, followed by 50 cycles at 95°C for 10 sec, 60°C for 20 sec and a cooling step to 40°C. To normalize the results obtained by qPCR, the expression of the GAPDH housekeeping gene was analyzed. Each sample was tested in triplicate. PCR efficiency for each gene was tested by serial dilutions of all genes. Amplification efficiencies of target genes and those of GAPDH were approximately equal.

### CASP3 protein ELISA

CASP3 family of proteases are key effectors in the apoptosis of mammalian cells. The measurement of CASP3 activity was determined using a luminescent assay according to the manufacturer’s instructions [PathScan^®^ Cleaved CASP3 (Asp175) Sandwich ELISA; Cell Signaling Technology, Inc.]. Cells were incubated with 25 ng/ml IL-6 for 3 h to detect the proapoptotic effects of the optimum doses (300 μM S3I-201; 50 μM AG490) of reagents alone and in combination with/without 100 ng/ml TRAIL. Adding the reagent to the wells resulted in cell lysis, followed by CASP cleavage of the substrate and generation of a luminescent signal produced by luciferase which is proportional to the amount of present CASP activity. The CASP3 activity was analyzed by reading the absorbance at 450 nm using a microplate ELISA reader (Tecan Austria GmbH, Grödig, Austria).

### DAPI/terminal deoxynucleotidyl transferase-mediated dUTP nick-end labeling (TUNEL) double staining assay

DNA fragmentation was detected by TUNEL, using the DeadEnd™ Fluorometric TUNEL System assay (Promega Corporation, Madison, WI, USA). Cells (5×10^3^) were plated into 96-well flat bottom plates (Corning Inc., Acton, MA, USA) and allowed to attach overnight prior to treatment with IL-6 (25 ng/ml), IL-6 + AG490 (50 μM), IL-6 + S3I-201 (300 μM), IL-6 + AG490 + TRAIL (25 ng/ml optimal effective dose) and IL-6 + S3I-201 + TRAIL for 24 h in fresh complete medium. The assay was performed as previously described by Kristjansdottir *et al* (35). Experiments were performed in triplicate.

### Statistical analysis

Statistical significance levels of differences in mRNA expression were analyzed by the pair-wise fixed reallocation randomization test. The REST software tool 2009 version 2.013 (36) was used for group-wise comparison and statistical analysis of relative expression results. Cell viability and alterations in apoptosis were analyzed using the one-way analysis of variance (ANOVA). Multiple comparison analysis was performed using SPSS version 15.0 (SPSS, Inc., Chicago, IL, USA). P<0.01 was considered to indicate a statistically significant difference. Data are expressed as the mean ± standard deviation.

## Results

### Cytotoxic activity

IL-6 (ranging between 1 and 100 ng/ml) treatment resulted in a dose- and time-dependent stimulation of LNCaP cell proliferation compared with the untreated controls. In the 25 ng/ml concentration of IL-6, increase in the cell viability was 20, 45 and 46% at 24, 48 and 72 h, respectively. Cell viability percentages for >25 ng/ml IL-6 were 21, 40 and 39% at 24, 48 and 72 h, respectively. In other words, the most profound effect of IL-6 was observed when cells treated with a 25 ng/ml concentration of IL-6 yielded a relatively higher cell viability than cells treated with concentrations of >25 ng/ml (data not shown).

The IC_50_ values of AG490 and S3I-201 were measured for the LNCaP cell line. Percentages of viability were 51.27, 51.54 and 52.62% at 24, 48 and 72 h, respectively, for a 50 μM concentration of AG490. On the other hand, percentages of viability were 52.27, 53.54 and 54.62% at 24, 48 and 72 h, respectively, for a 300 μM concentration of S3I-201. The IC_50_ values were verified by WST-1 assay when the cells in culture were treated with 25 ng/ml IL-6 alone and combinations of IL-6 + AG490 and IL-6 + S3I-201 for all incubation periods.

The addition of TRAIL *per se*, did not affect the percentage of the cell viability even when the cells were treated with the highest concentration (1,000 ng/ml). The addition of TRAIL to the combination [IL-6 (25 ng/ml) + AG490 (50 μM) + TRAIL (100 ng/ml; mean value of TRAIL concentrations)] did not cause a significant change as cell viability percentages were 49, 50 and 51.2% at 24, 48 and 72 h, respectively. Furthermore, when the combination of IL-6 + S3I-201 (300 μM) + TRAIL was used, cell viability percentages were 53.42, 53.32 and 52.26% at 24, 48 and 72 h, respectively (data not shown).

### AG490 and S3I-201 inhibit IL-6-induced STAT3 phosphorylation

**Figure 1:**
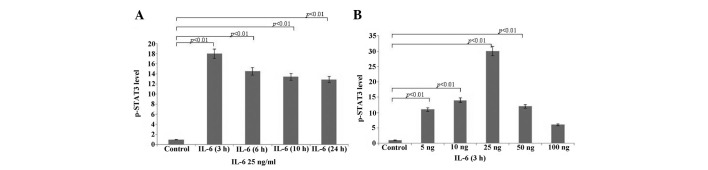
(A) Variations in p-STAT3 protein levels in LNCaP cells treated with 25 ng/ml IL-6 at various time periods were quantified by ELISA. (B) ELISA analysis of dose-dependent variations in p-STAT3 protein levels in LNCaP cells treated with IL-6 for 3 h. STAT3, signal transducer and activator of transcription 3; IL-6, interleukin-6; ELISA, enzyme-linked immunosorbent assay.

To determine whether AG490 and S3I-201 inhibit p-STAT3 (STAT3 activation) human LNCaP cells which do not constitutively express activated STAT3, IL-6 was exogenously added to induce the phosphorylation of STAT3 at Tyr705. Firstly, to confirm the most effective dose of IL-6, dose and time changes were measured in the level of p-STAT3 by ELISA assay. The highest level of p-STAT3 was found following 3 h of incubation with IL-6, which resulted in an 18-fold increase in comparison with the control (P<0.01; Fig. 1A). Among the doses studied, the highest level of p-STAT3 which indicated a 30-fold increase (Fig. 1B; P<0.01) was observed at 25 ng/ml IL-6 treatment. Therefore, the dose previously found to be most effective by WST-1 assay was confirmed.

**Figure 2:**
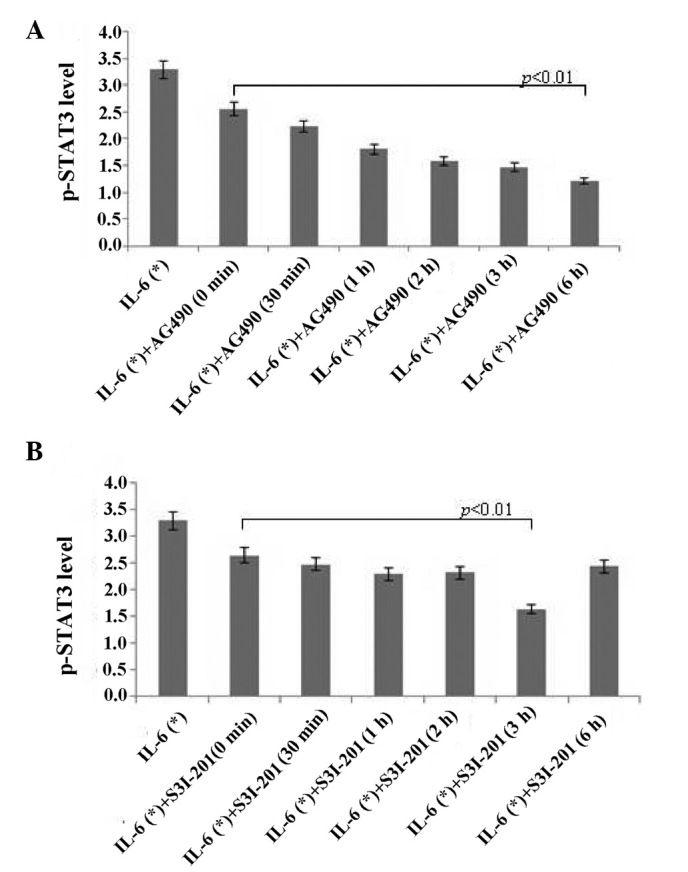
Variations in p-STAT3 protein levels in LNCaP cells treated with IL-6 followed by various durations of treatment with (A) AG490 (50 μM) and (B) S3I-201 (300 μM). (*)Cells previously treated with 25 ng/ml IL-6 for 3 h. STAT3, signal transducer and activator of transcription 3; IL-6, interleukin-6.

Following 3 h of incubation with IL-6, the cells were treated with various doses of AG490 and S3I-201 inhibitors. Among the doses studied, the lowest level of p-STAT3 was observed at 50 μM AG490 and 300 μM S3I-201 (data not shown). The doses previously found by WST-1 assay were verified. Treatment with combinations of IL-6 + AG490 and IL-6 + S3I-201 for the indicated time periods led to the lowest level of p-STAT3 at 6 h with 50 μM AG490 and 3 h with 300 μM S3I-201 (Fig. 2; P<0.01).

**Figure 3:**
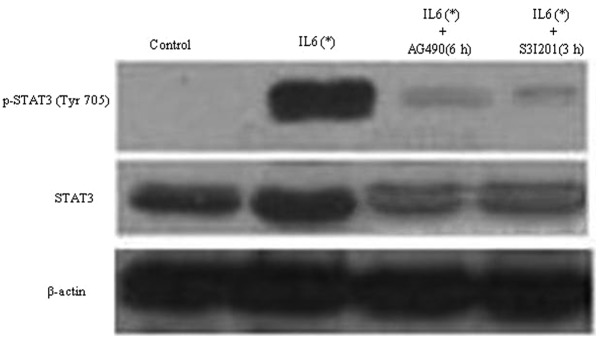
Representative western blot analysis showing that AG490 (50 μM) and S3I-201 (300 μM) suppressed the tyrosine phosphorylation of STAT3 by IL-6. For suppression effect, cells were stimulated with IL-6 for 3 h and then with AG490 and S3I-201 for 6 and 3 h, respectively. The levels of β-actin expression were set as a control for equivalent protein loading. (*)Cells previously treated with 25 ng/ml IL-6 for 3 h. STAT3, signal transducer and activator of transcription 3; IL-6, interleukin-6.

Compared with the control cells, western blot analysis revealed that p-STAT3 and STAT3 protein levels in LNCaP cells were significantly downregulated following treatment with combinations of IL-6 (3 h; 25 ng/ml) + AG490 (6 h; 50 μM) and IL-6 (3 h; 25 ng/ml) + S3I-201 (3 h; 300 μM). Levels of β-actin remained unchanged in response to treatment with inhibitors (Fig. 3).

### AG490 and S3I-201 inhibit JAK/STAT3-mediated gene expression

**Figure 4:**
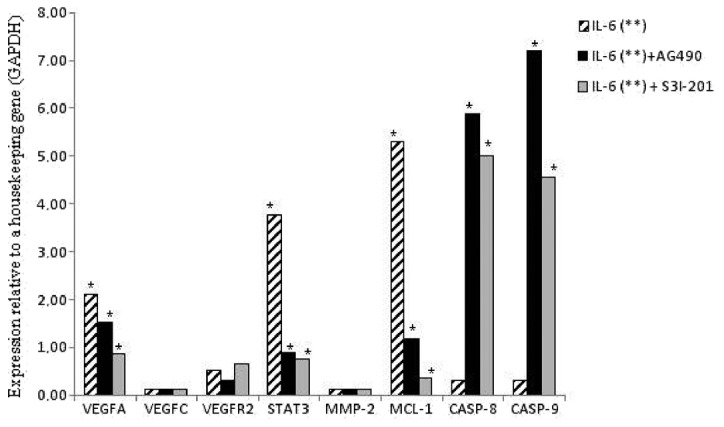
Effects of AG490 and S3I-201 on mRNA levels of the genes in the LNCaP cell line. Real-time polymerase chain reaction was performed using specific primers for VEGFA, VEGFC, VEGFR2, STAT3, MMP-2, MCL-1, CASP8 and CASP9 with total RNA isolated from the prostate cancer LNCaP cell line. ^*^P<0.01, vs. IL-6 only. (*)Cells previously treated with 25 ng/ml IL-6 for 3 h. VEGFA, vascular endothelial growth factor A; VEGFC, vascular endothelial growth factor C; VEGFR2, vascular endothelial growth factor receptor 2; STAT3, signal transducer and activator of transcription 3; MMP-2, matrix metalloproteinase-2; MCL-1, myeloid cell leukemia sequence 1; CASP8, caspase 8; CASP9, caspase 9; IL-6, interleukin-6.

Alterations of gene expression in LNCaP cells treated with IL-6 alone and combinations of IL-6 (3 h; 25 ng/ml) + AG490 (6 h; 50 μM) and IL-6 (3 h; 25 ng/ml) + S3I-201 (3 h; 300 μM) at the mRNA level were verified for all selected genes. It was shown that the results for these genes were consistent with the apoptosis and cell proliferation inhibition analysis. Following treatment with combinations of IL-6 + AG490 and IL-6 + S3I-201 for 24 h, statistically significant differences were identified in the mRNA expression levels of the VEGFA, STAT3, MCL-1, CASP8 and CASP9 genes when compared with sole IL-6 treatment (Fig. 4; P<0.01). As shown in Fig. 4, use of 25 ng/ml IL-6 alone resulted in an increase in STAT3, VEGFA and MCL-1, but a decrease in CASP8 and CASP9. Notably, the VEGFC and MMP-2 mRNA levels in LNCaP cells were found to be extremely low. Furthermore, no differences were observed in the mRNA levels of VEGFR2 between the sole IL-6 treatment group and treatment with combinations of IL-6 + AG490 and IL-6 + S3I-201 for 24 h (P>0.05). In the presence of the two inhibitors, gene expression of MCL-1 decreased significantly compared with the IL-6 alone group (P<0.01). Treatment with combination of IL-6 + 50 μM AG490 resulted in a decrease in STAT3 (4.22-fold), VEGFA (1.39-fold) and MCL-1 (4.47-fold) at 24 h. Treatment with combination of IL-6 + 300 μM S3I-201 resulted in a decrease in STAT3 (5.01-fold), VEGFA (2.41-fold) and MCL-1 (14.66-fold) at 24 h. These decreases were greater than the decreases observed following treatment with combination of IL-6 + 50 μM AG490 at 24 h. By contrast, treatment with combination of IL-6 + 50 μM AG490 resulted in an increase in CASP8 (18.93-fold) and CASP9 (23.25-fold) at 24 h. Treatment with combination of IL-6 + 300 μM S3I-201 also resulted in an increase in CASP8 (16.12-fold) and CASP9 (14.74-fold) at 24 h. These results suggested that AG490 and S3I-201 regulate the expression of specific genes involved in cell growth, angiogenesis and apoptosis at the mRNA level in a dose- and time-dependent manner.

### AG490 and S3I-201 promotes IL-6-induced activation of CASP3

**Figure 5:**
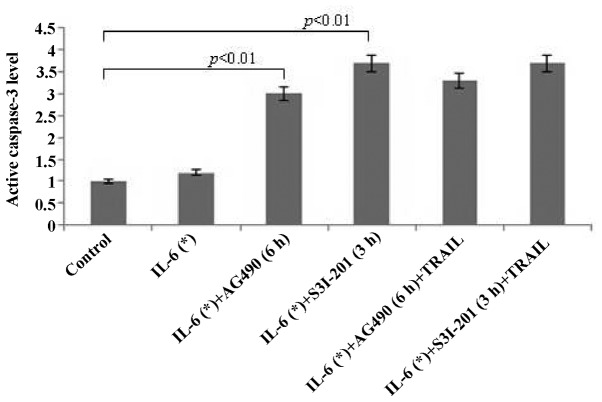
AG490 and S3I-201 enhance IL-6-induced activation of caspase activity in human prostate cancer cells. LNCaP prostate cancer cells were treated with the indicated concentrations of AG490 and S3I-201 alone and in combination with TRAIL (100 ng/ml) for 24 h and the activities of caspase-3 were examined by enzyme-linked immunosorbent assay. (*)Cells previously treated with 25 ng/ml IL-6 for 3 h. IL-6, interleukin-6; TRAIL, tumour necrosis factor-related apoptosis-inducing ligand.

The activity level of CASP3 (Fig. 5) greatly increased in LNCaP cells exposed to combinations of IL-6 (3 h; 25 ng/ml) + AG490 (6 h; 50 μM) and IL-6 (3 h; 25 ng/ml) + S3I-201 (3 h; 300 μM) for 24 h (P<0.01). These results indicated that AG490 and S3I-201 promote IL-6-induced apoptosis via CASP-dependent apoptotic pathways in prostate tumor cells. No statistically significant differences were identified in the CASP3 expression levels between treatment with inhibitors alone and combinations of inhibitor + TRAIL (Fig. 5).

### TUNEL analysis of apoptotic cells

**Figure 6:**
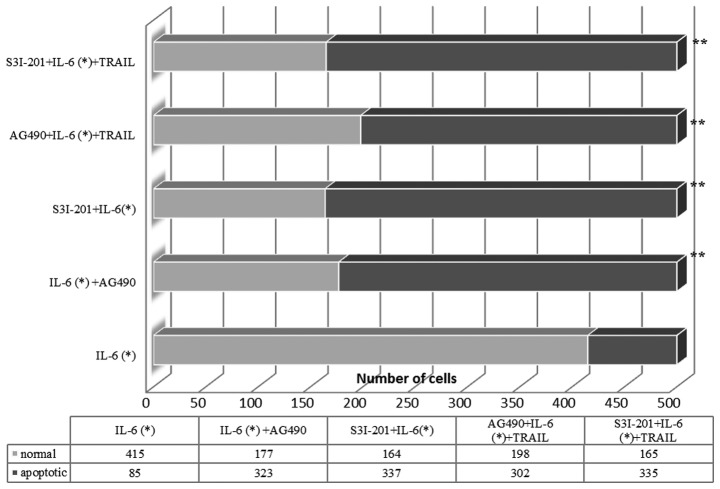
AG490 and S3I-201 augmented IL-6-induced apoptosis in human prostate cancer cells as detected by TUNEL assay. LNCaP cells were exposed to the indicated concentrations of IL-6 + AG490 and IL-6 + S3I-201 and in combination with TRAIL (100 ng/ml) for 24 h and apoptosis was determined by a TUNEL assay kit. (*)Cells previously treated with 25 ng/ml IL-6 for 3 h and ^**^P<0.01, vs. IL-6 only. IL-6, interleukin-6; TUNEL, terminal deoxynucleotidyl transferase-mediated dUTP nick-end labeling; TRAIL, tumor necrosis factor-related apoptosis-inducing ligand.

To validate whether AG490 and S3I-201 enhance IL-6-induced activation of CASP activity in human CaP cells, TUNEL assay was used to quantify the effects of the inhibitors on the number of IL-6-induced apoptotic cells. The number of apoptotic cells following treatment with combinations of IL-6 (3 h; 25 ng/ml) + AG490 (6 h; 50 μM) and IL-6 (3 h; 25 ng/ml) + S3I-201 (3 h; 300 μM) for 24 h were ~4-fold higher than the number of apoptotic cells following treatment with IL-6 alone (Fig. 6). However, addition of TRAIL to the combinations did not affect the number of apoptotic cells. An independent Giemsa analysis of apoptotic cells revealed similar quantitative results (data not shown).

## Discussion

The JAK/STAT3 signaling pathway is crucial in regulating a number of pathways in tumorigenesis. During malignant transformation, STAT3 is frequently overexpressed and constitutively activated by tyrosine phosphorylation. Certain studies have previously shown that activated STAT3 is overexpressed in cancer tissues and cell lines (37–40). Despite the apparent importance of STAT3 in cell proliferation, metastasis and survival in CaP, its potential molecular mechanisms involving CaP tumorigenesis have not been entirely characterized. This may yield promising results for the future of cancer in terms of adjuvant cancer therapies involving IL-6-induced LNCaP in human CaP cells. Due to the pleiotropy of IL-6, the outcome of this cytokine-targeting therapy may be unpredictable in CaP patients, ranging from a lack of response to beneficial or detrimental effects (41). Therefore, the present study focused on AG490 and S3I-201 inhibitors of the JAK/STAT3 signaling pathway.

In LNCaP cells where STAT3 is not constitutively active, the exogenous addition of IL-6 induced the phosphorylation of STAT3 at Tyr705 which had been reduced by JAK/STAT3 inhibitors. AG490 is a well-known JAK2 inhibitor which is able to effectively block STAT3 activation in different cancer cell lines (42–44). On the other hand, NSC 74859, one of the novel STAT3 inhibitors, significantly suppresses tumor growth *in vitro* and *in vivo* (45,46). The aim of the current study was to determine whether AG490 and S3I-201 are effective in inhibiting STAT3 phosphorylation in LNCaP cells. This effect may modulate the expression of a number of the well-known angiogenic and apoptotic/antiapoptotic genes. It was revealed that the addition of AG490 (50 μM) and S3I-201 (300 μM) to the IL-6-stimulated cells resulted in suppression of the tyrosine phosphorylation of STAT3 in a time-dependent manner. Such downregulation was paralleled by an evident decrease of p-STAT3 protein expression. p-STAT3 protein levels were found to be significantly lower in cells treated with AG490 and S3I-201, compared with the IL-6-stimulated cells. Western blot analysis showed weak expression levels (the intensity of immunoreactivity bands) of STAT3 protein when treated with inhibitors. Nevertheless, no change in the level of STAT3 was observed following stimulation with IL-6 only compared with the control, which suggested that the upregulation of p-STAT3 may be attributed to the activation of upstream factors. Previous studies have demonstrated that the AG490 does not affect the total protein levels of STAT3 (12,37). Notably, in contrast to this, the present study found that STAT3 protein levels in LNCaP cells were significantly downregulated following treatment with AG490 (50 μM) and S3I-201 (300 μM). The differences between the observations of the current study and those of Seo *et al* (12) and Huang *et al* (37) stem from various factors, including incubation periods and instructions, the different molecular mechanisms used to induce apoptosis on the various types of cultured cells and laboratory conditions. In healthy human and animal cells, ligand-dependent activation of STATs is a transient process, lasting for several minutes to several hours. By contrast, the results of the current study are consistent with those of a previous study by Zhang *et al* (19). Treatment of hepatoma cell lines with NSC 74859 resulted in the downregulation of p-STAT3 levels, whereas no change was identified in the levels of total STAT3 (46). A novel small molecule, LLL12, which targets STAT3, was found to inhibit STAT3 phosphorylation and induce apoptosis in various breast, pancreatic and glioblastoma cancer cell lines (47). Downstream targets of STAT3, cyclin D1, Bcl-2 and survivin, were also downregulated by LLL12 at the protein and mRNA levels. The results of the present study with regard to S3I-201 are consistent with those of a previous study by Lin *et al* (47). It was not possible to compare the results of the current study with those of other studies as no previous studies have analyzed the protein expression levels of STAT3 and p-STAT3 following IL-6 + AG490 and IL-6 + S3I-201 administration in LNCaP cells. The present study has demonstrated that AG490 and S3I-201 markedly suppress STAT3 activity and that IL-6 promotes STAT3 activity in LNCaP cell lines.

Inappropriate activation of STAT3 may be responsible for CaP progression by regulating the expression of angiogenic and apoptotic/antiapoptotic genes. The functional inactivation of STAT3 by inhibitors may inhibit cell proliferation and promote the apoptosis of CaP cells. In the present study, the use of AG490 and S3I-201 markedly reduced VEGFA, STAT3 and MCL-1 mRNA expression in LNCaP cells. The regulatory mechanism for MCL-1 expression in CaP cells remains elusive due to the limited information available on its expression profile. AG490-induced apoptosis in leukemic large granular lymphocytes was independent of Bcl-xL or Bcl-2 expression. However, Epling-Burnette *et al* (48) previously found that the Bcl-2 family protein, MCL-1, was significantly reduced by AG490 treatment. Results of the current study demonstrated that induction of apoptosis involved the inhibition of STAT3 and p-STAT3 activity by downregulating the expression of a known STAT3 target gene, MCL-1. The decrease in the transcriptional expression level of the MCL-1 gene confirms that LNCaP cells are directed toward apoptosis. In the present study, LNCaP cells expressed the mRNA of VEGFA but not of VEGFC. A previous study by Zhang *et al* (33) detected no VEGFR1 expression in ARCaP_M_, ARCaP_M_-C_2_, PC_3_, LNCaP, C_4–2_ and C_4–2_ B cell lines, but found a low expression level for VEGFR2 in only one cell line (ARCaP_M_-C_2_). However, it remains controversial whether VEGF exhibits significant autocrine effects in CaP cells, since the ‘classical’ VEGFRs, i.e., VEGFR1 and VEGFR2, are undetectable in the majority of established CaP cell lines (49). The results of the current study are in line with those of a previous study by Zhang *et al* (33) which exhibited the low expression of VEGFR2 in LNCaP cells. These results suggest that neuropilin-1 may be the major receptor mediating VEGF effects in CaP cells. Previously, it has been shown that AG490 markedly inhibits angiotensin II-induced STAT3 activation and the expression of MMP-2 and VEGF in gastric cancer cells (50). Furthermore, the use of AG490 markedly reduced MMP-2 mRNA expression in human pancreatic cells (SW1990) (37). However, the current study unexpectedly found no MMP-2 and VEGFC mRNA expression in LNCaP cells. CASPs are the central executors of the apoptotic process and CASP3, CASP8 and CASP9 are considered to be markers of different apoptotic pathways. CASP8 and CASP9 expression at mRNA level was investigated to determine the efficacy of AG490 and S3I-201 via the CASP cascade-mediated pathway in LNCaP cells. For CASP8 and CASP9, the increase in the mRNA expression levels in comparison with the controls (treated with IL-6 alone) was found to be statistically significant (P<0.01). Again, it was not possible to compare the results of the present study with those of other studies as no previous studies have analyzed the mRNA expression levels of CASP8 and CASP9 following the administration of inhibitors to LNCaP cells. The mRNA results of the current study showed that IL-6 + AG490 treatment is more effective than the IL-6 + S3I-201 treatment in terms of leading the LNCaP cells to apoptosis. On the other hand, IL-6 + S3I-201 treatment plays a regulatory role on the angiogenic and antiapoptotic genes. The two treatments may trigger apoptosis in cells through the extrinsic or intrinsic pathways. A decrease in the mRNA level of STAT3 following the two treatments led to the hypothesis that these inhibitors may affect the phosphorylation of STAT3 and more directly the STAT3 gene itself. Similar results were achieved for the active CASP3 protein level which revealed that AG490 and S3I-201 caused an increase in the CASP3 protein level. This may be attributed to activation of the apoptotic signaling pathway. To validate these results, TUNEL assays were used to quantify the effect of AG490 and S3I-201 with/without TRAIL on the number of cells previously treated with IL-6. The observations suggested that the cells are directed towards apoptosis upon administration of inhibitors alone and with combinations of TRAIL. In addition, the observations were consistent with those of other studies which have explored different cell lines (8,47).

Lanuti *et al* (43) previously reported that the combinatorial effect of TRAIL and AG490 on T-cell leukemia was characterized by a significant inhibition of STAT3 phosphorylation compared with the controls or TRAIL alone-treated samples (43). However, cell viability, CASP3 activity and TUNEL assay results of the present study showed that LNCaP cells are resistant to TRAIL-induced apoptosis. There are a number of possible causes of resistance to TRAIL as follows: higher expression of cellular IAP proteins (51); interference of the phosphatidylinositide 3-kinase/Akt pathway with an apoptotic signal by inhibiting the processing of Bid (52); and TRAIL receptor DR4 protein degradation by the ubiquitin-proteasome (53).

The current study focused on IL-6 + AG490 and IL-6 + S3I-201 treatments but not on the combined IL-6 + AG490 + S3I-201 treatment due to the evidence that each inhibitor affects the JAK/STAT pathway during different phases. In addition, the trials involving the latter combination did not provide comparatively significant results in terms of leading the cells to apoptosis (data not shown).

The observations of the present study shed light on a novel biological effect produced by the chemotherapeutic drugs, AG490 and S3I-201. To the best of our knowledge, the study shows for the first time that the selective JAK2 inhibitor, AG490 and STAT3 inhibitor, S3I-201, downregulate MCL-1, STAT3 and VEGFA and that this process is accompanied by the decreased proliferation and increased apoptosis of LNCaP cancer cells. In addition, it was shown that VEGFA exhibits a primary role in the JAK/STAT3 pathway and that its activation is not dependent on VEGFR2. These inhibitors may be effective target molecules for antiangiogenic therapy. Future treatments that combine antiangiogenic treatments with conventional therapy may lead to increased clinical efficacy for the benefit of CaP patients. These observations may aid the development of new anticancer strategies. Since preclinical results have been promising, additional animal model studies must be undertaken to determine whether overexpression of the JAK/STAT pathway is ‘driving’ prostate carcinogenesis.

In conclusion, the current study holds therapeutic promise for JAK/STAT inhibitors for the treatment of CaP by targeting constitutively activated STAT3. The JAK/STAT3 signaling pathway is likely to be an important mediator in the pathogenesis of CaP. Therefore, we hypothesized that the future use of the JAK2- and STAT3-specific inhibitors may be a new approach in blocking the development of CaP.

## Figures and Tables

**Table I:** Gene-specific primer sequences and probe numbers.

Gene probe	Forward primer	Reverse primer	UPL
GAPDH	5′-AGCCACATCGCTCAGACAC-3′	5′-GCCCAATACGACCAAATCC-3′	60
VEGFA	5′-AGTGTGTGCCCACTGAGGA-3′	5′-GGTGAGGTTTGATCCGCATA-3′	9
VEGFC	5′-TGCCAGCAACACTACCACAG-3′	5′-GTGATTATTCCACATGTAATTGG-3′	27
VEGFR2	5′-GAACATTTGGGAAATCTCTTGC-3′	5′-CGGAAGAACAATGTAGTCTTTGC-3′	18
STAT3	5′-CCCCGCACTTTAGATTCATT-3′	5-CATGTCAAAGGTGAGGGACTC-3′	18
MMP-2	5′-CCCCAAAACGGACAAAGAG-3′	5′-CTTCAGCACAAACAGGTTGC-3′	43
MCL-1	5′-AAGCCAATGGGCAGGTCT-3′	5′-TGTCCAGTTTCCGAAGCAT-3′	4
CASP8	5′-TCCAAATGCAAACTGGATGA-3′	5′-TCCCAGGATGACCCTCTTCT-3′	62
CASP9	5′-CCATATGATCGAGGACATCCA-3′	5′-GACTCCCTCGAGTCTCCAGAT-3′	27

UPL, Universal Probe Library; VEGFA, vascular endothelial growth factor A; VEGFC, vascular endothelial growth factor C; VEGFR2, vascular endothelial growth factor receptor 2; STAT3, signal transducer and activator of transcription 3; MMP-2, matrix metalloproteinase-2; MCL-1, myeloid cell leukemia sequence 1; CASP8, caspase 8; CASP9, caspase 9.
